# Behavioural coordination of dogs in a cooperative problem-solving task with a conspecific and a human partner

**DOI:** 10.1007/s10071-013-0676-1

**Published:** 2013-09-01

**Authors:** Ljerka Ostojić, Nicola S. Clayton

**Affiliations:** Department of Psychology, University of Cambridge, Cambridge, CB2 3EB UK

**Keywords:** Dogs, Cooperative problem-solving, Domestication, Behavioural coordination

## Abstract

The process of domestication has arguably provided dogs (*Canis familiaris*) with decreased emotional reactivity (reduced fear and aggression) and increased socio-cognitive skills adaptive for living with humans. It has been suggested that dogs are uniquely equipped with abilities that have been identified as crucial in cooperative problem-solving, namely social tolerance and the ability to attend to other individuals’ behaviour. Accordingly, dogs might be hypothesised to perform well in tasks in which they have to work together with a human partner. Recently, researchers have found that dogs successfully solved a simple cooperative task with another dog. Due to the simplicity of the task, this study was, however, unable to provide clear evidence as to whether the dogs’ successful performance was based on the cognitive ability of behavioural coordination, namely the capacity to link task requirements to the necessity of adjusting one’s actions to the partner’s behaviour. Here, we tested dogs with the most commonly used cooperative task, appropriate to test behavioural coordination. In addition, we paired dogs with both a conspecific and a human partner. Although dogs had difficulties in inhibiting the necessary action when required to wait for their partner, they successfully attended to the two cues that predicted a successful outcome, namely their partner’s behaviour and the incremental movement of rewards towards themselves. This behavioural coordination was shown with both a conspecific and a human partner, in line with the recent findings suggesting that dogs exhibit highly developed socio-cognitive skills in interactions with both humans and other dogs.

**Electronic supplementary material:**

The online version of this article (doi:10.1007/s10071-013-0676-1) contains supplementary material, which is available to authorized users.

## Introduction

Cooperative problem-solving is required when a task cannot be solved by one individual alone and where successful performance relies on at least two individuals working together (Chalmeau and Gallo 1996; Visalberghi 1997; Schuster and Perelberg 2004). Social species, especially those that participate in group activities such as group hunting, are hypothesised to have evolved cognitive mechanisms that enable them to flexibly and efficiently solve problems with other individuals. For example, in the benchmark test of cooperative problem-solving, the ‘loose string’ task (Melis et al. 2006a, b; Hirata and Fuwa 2007; Seed et al. 2008; Péron et al. 2011; Plotnik et al. 2011), both individuals need to pull the string ends simultaneously to obtain the food reward. If only one individual pulls, the string will become unattached from the apparatus and neither subject will obtain the reward. A dyad’s performance in this cooperative task therefore depends on the individuals’ ability to link the necessity of the partner to the task requirement and attend to each other in order to adjust one’s own pulling action to the behaviour of the partner (Chalmeau and Gallo 1996). Note that successful performance in this task does not, however, depend on ‘joint action’, in that individuals need not know that their partner shares with them a common goal and a shared intention to achieve this goal (Waneken et al. 2006). Successful performance in the current cooperative problem-solving tasks designed for non-human animals can therefore be solved by mechanisms such as associative learning, as long as individuals correctly identify the social stimulus of their partner’s behaviour as instrumental in solving the task and coordinate their own actions accordingly (Seed and Jensen 2011).

This cognitive ability of behavioural coordination is commonly assessed using a delay task, in which one of the individuals is delayed in approaching the apparatus such that their partner needs to wait for them and inhibit performing the necessary action until the other subject is ready to participate in the task (Melis et al. 2006b; Seed et al. 2008; Péron et al. 2011; Plotnik et al. 2011). Following an initial training to facilitate inhibition of the required response, both chimpanzees (*Pan troglodytes*; Melis et al. 2006b) and Asian elephants (*Elephas maximus*; Plotnik et al. 2011) generalised this behaviour onto novel, longer delays of the partner.

In addition to behavioural coordination, a further non-cognitive factor has been identified as an important influence in solving cooperative tasks, namely inter-individual tolerance. Several studies report that more tolerant individuals outperformed less tolerant ones (chimpanzees: Chalmeau and Gallo 1993; Melis et al. 2006a; rooks (*Corvus frugilegus*): Seed et al. 2008; Scheid and Noë 2010). In addition, bonobos (*Pan paniscus*), whose social system is characterised by high levels of social tolerance, were more successful in solving a cooperative task than the less tolerant chimpanzees when the rewards could easily be monopolised by one individual (Hare et al. 2007). In summary, two factors appear to act as constraints on cooperative problem-solving: firstly, the motivation to be close to other individuals even around food (social tolerance) and, in addition, the ability to attend to other individuals’ behaviour and adjust one’s own actions accordingly (behavioural coordination).

Due to their history of domestication, domestic dogs (*Canis familiaris*) might be uniquely equipped with the motivational and cognitive abilities that facilitate cooperative problem-solving. Dogs evolved from wolves (*Canis lupus*; Vila et al. 1997; Galibert et al. 2011), and through the process of domestication, they became adapted to a niche created by humans. Archaeological and phylogenetical data highlight the cooperative and tolerance-based relationship between human and dogs throughout the history of domestication (Nobis 1979; Clutton-Brock 1984, 1995; Coppinger and Coppinger 2001; Morey 2006). Thus, the evolution of dogs’ cognitive abilities may be closely linked to cooperation, a relationship that is thought to have uniquely shaped human cognition and culture (Tomasello 1999, 2007; Warneken and Tomasello 2006; Herrmann et al. 2007; Moll and Tomasello 2007). Two non-mutually exclusive hypotheses have been put forward regarding the phenotypic changes during domestication. Firstly, dogs are thought to have a decreased emotional reactivity compared to their ancestors (Clark and Ehlinger 1987; Hare and Tomasello 1999). Lower levels of fear and aggression towards both conspecifics and humans than seen in wolves (Miklósi 2009) would have allowed close contact to humans and thus the development of close social bonds (Hare and Tomasello 1999). Secondly, domestication is thought to have specifically selected for socio-cognitive abilities to facilitate dog–human interactions (Miklósi et al. 2004; Csányi 2005; Kubinyi et al. 2007; Miklósi 2009). Such socio-cognitive abilities are shown both in the flexibility of dogs to *produce* visual and acoustic signals, arguably to communicate with humans (Schassburger 1993; Yin 2002; Pongrácz et al. 2005; Molnár et al. 2008), and in their *ability to respond to* such signals when produced by humans (use of visual signals: Miklósi et al. 2000, 2003; Hare et al. 2002; Virányi et al. 2004, 2006; Riedel et al. 2008; Kupán et al. 2011; use of acoustic signals: McConnell 1990; Kaminski et al. 2004).

Despite extensive research on dogs’ temperamental factors and socio-cognitive abilities, there is a lack of studies investigating dogs’ performance in cooperative problem-solving tasks, in which one individual alone cannot achieve success and the individual's behaviours are aimed at solving a specific physical problem by working with another individual. A notable exception is a recent study in which pairs of dogs were tested on two tasks that required them to inhibit approaching food through an initially open door and instead approach an initially closed door (Bräuer et al. 2013). Although dogs were successful in performing this behaviour and thus obtaining the food reward, the tasks could be solved by the dogs following a strategy without attending to each other. Thus, it remains unclear whether the dogs’ successful performance was due to spatial and temporal adjustment of behaviours (coordinated action) or due to learning a rule that did not involve a sensitivity to the social cue of the partner’s behaviour. Bräuer et al. (2013) therefore propose that future studies will benefit from using a more complex task that requires behavioural coordination. Ideally, such a task would involve a training phase in which individuals can learn how the task is solved by themselves (‘alone’ version), before being presented with a cooperative version of the task together with a partner. Importantly, there needs to be a contingency between the physical properties of the task between the training and test phases, such that the only factor that changes is the necessity of a partner (see Melis et al. 2006a, b and Seed et al. 2008 for successful implementation of such a contingency).

Several experiments showed that dogs have difficulties solving tasks requiring knowledge of physical causalities (Bräuer et al. 2004; Osthaus et al. 2005; Udell et al. 2008). Consequently, dogs may be unable to learn the contingencies of the physical aspects of a cooperative problem-solving task. However, a recent study found that dogs were sensitive to physical causalities and successfully solved a means-end task (Range et al. 2011), suggesting that testing them on cooperative tasks with a higher physical complexity should not be excluded a priori.

In the current study, we tested dogs on one of the most commonly used cooperative problem-solving paradigms, namely the ‘loose string’ task (Melis et al. 2006a, b; Hirata and Fuwa 2007; Seed et al. 2008; Péron et al. 2011; Plotnik et al. 2011). In this task, food can be obtained by pulling on two string ends simultaneously. Initially, the dogs had to learn how to solve this physical task by themselves using an ‘alone’ version of the apparatus in which the string ends were close enough to both be pulled by a single individual. Importantly, a transfer test was conducted at the end of the training phase to ensure that dogs could generalise the learnt rule of having to pull both string ends at the same time to novel situations. Subsequently, they were given the cooperative version of the task in which the string ends were too far apart for one individual to solve the task alone. To assess whether their performance was based on coordinated actions, dogs were then presented with a delay task in which one of the partners had to overcome a physical obstacle and was thus delayed in their approach.

Dogs were paired both with a conspecific and with a familiar human partner. There are two reasons why dogs might be expected to perform better with a human partner. Firstly, dogs might be more used to attend to their owner’s behaviour rather than that of another dog in a problem-solving context. Secondly, if domestication has selected specifically for socio-communicative abilities towards humans, then dogs might find it easier to identify the human partner’s behaviour as instrumental in solving the task. Such a result would also support the idea that domestication has led to the emergence of representations of social agents that are specific to dog–human interactions.

## Methods

### Subjects

Twenty-nine dogs of different breeds and ages participated in the experiments and were tested in Croatia between July and October in 2010 and 2011. Owners and their dogs were recruited through dog schools and the Croatian Rescue and Search Dog Association (CRDA). Thus, all dogs were trained in basic commands, and in addition, some of them were trained as Search and Rescue dogs whilst others received some training in agility (see Table 1). Only eleven dogs (one male and ten females; age range 2–12 years at the onset of testing) completed the experiment (see Table 1); the other dogs either did not successfully complete the training stage (training was aborted if dogs did not make any progress on the first training phase within three testing sessions and showed no interest in the apparatus—3 dogs in total), or their owners did not have enough time for the dogs to participate in the whole experiment (15 dogs in total). Therefore, all dogs that successfully solved the first training phase and subsequently did not drop out due to their owners successfully passed all training phases and proceeded to the testing stage of our study. To prevent aggression, dog–dog dyads were formed exclusively by dogs that lived in multiple-dog households and were thus familiar with each other. This procedure also ensured that dogs participating in the dog–dog dyads were used to attend to other dogs as well as humans in their daily lives. Dog–human dyads were formed by the experimenter and dogs from both one-dog and multiple-dog households. One dog participated in the dog–dog experiment after having participated in the dog–human one (Ska). Two dogs participated in two different dyads in the dog–dog experiment (Sapa and Rama). All testing was carried out either inside the owner’s house or in an outdoor area familiar to the dog, such as the owner’s garden or a familiar training area. All owners were instructed not to feed their dogs 2 h prior to testing. Pieces of ham were used as rewards, and dogs had unrestricted access to bowls of water throughout testing. The experiments were approved by the University of Cambridge.

**Table 1:** Dogs participating in training and testing

Dog’s name	Breed	Sex	Working dog
*Dogs participating in training*			
Charlie	Golden retriever	M	No
Sapa	Labrador retriever	F	Search and rescue
Keito	German hunting terrier	F	Search and rescue
Suky	German hunting terrier	F	Search and rescue
Rama	Labrador retriever	F	Search and rescue
Ska	Labrador retriever	F	Search and rescue
Chilli	Parson Russell terrier	F	Agility
Zara	Mixed breed	F	No
Lady	Mixed breed	F	Agility
Anouk	Parson Russell terrier	F	No
Onna	Groenendael	F	Search and rescue
Svrco	Mixed breed	M	No
Hara	Golden retriever	F	No
Moro	Mixed breed	M	Search and rescue
Timi	Mixed breed	M	Search and rescue
Buks	Golden retriever	M	Search and rescue
Pink	Parson Russell terrier	F	No
Luksa	Malinois	F	Search and rescue
Tau	Malinois	M	Search and rescue
Nera	Mixed breed	F	Search and rescue
Don	Labrador retriever	M	Search and rescue
Lars	Labrador retriever	M	Search and rescue
Ares	American Staffordshire terrier	M	Search and rescue
Abba	Labrador retriever	F	Search and rescue
Rem	Mixed breed	M	Search and rescue
Andrej	Parson Russell terrier	M	No
Bambi	Parson Russell terrier	F	Agility

Dog–dog dyads	Dog–human dyads
*Dogs participating in testing*	
Charlie and Sapa	Ska
Rama and Keito	Anouk
Lady and Chilli	Keito
Ska^^^ and Sapa*	Suky
Onna and Rama*	Zara

*M* male, *F* female dogs

* Dogs that participated in the experiment in a dog–dog dyad for the second time

^^^Dogs that participated in the experiment in the dog–human dyad first

### Apparatus

**Fig. 1:**
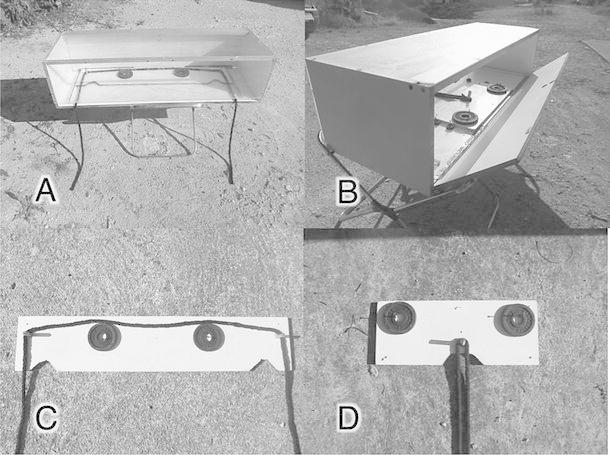
**a** Testing apparatus from the front. A box with a clear Perspex top and front was positioned on stools. At the front, the Perspex had an aperture. Inside the *box*, the experimenter positioned a platform with weights (black circles in the picture) and cylinders as well as notches in which food could be placed. A rope was positioned around the cylinders, and the rope ends were hung through the aperture at the front of the box. By pulling on both rope ends, the platform could be pulled forward such that the food fell on the ground. The weights were used because without them the platform was too light and thus pulling on one rope end alone produced enough friction to pull the platform into reach. **b** Testing apparatus from the back. The back of the box could be opened by the experimenter to position the platform, rope and food. **c** The ‘cooperation’ platform with two notches in which food could be placed, two weights (black round circles in the picture), two cylinders and a rope that was placed around them. This platform was used in the test phase of the experiment. **d** The ‘alone’ platform with one notch in which food could be placed, two weights (black round circles in the picture), one cylinder and a rope that was placed around it. This platform was used during the training phase and the transfer task at the end of training

The task was based on the ‘cooperation’ apparatus previously used with chimpanzees (Hirata and Fuwa 2007) and rooks (Seed et al. 2008). A box (120 × 41 × 30 cm) containing a platform was positioned on one (‘cooperation’ platform) or two (‘alone’ platform) stools, 37 cm above the ground (Fig. 1a, b). A ‘loose’ rope was threaded through two moving cylinders with both ends reaching outside the box through a 3-cm-high aperture at the front (see Fig. 1a). The platform contained notches in which food was placed and dogs could pull out the platform and access the food by pulling on both ends of the rope simultaneously. The ‘alone’ platform (50 × 1 × 20 cm) with one notch and the ‘close ends’ rope (187 cm long) could be successfully pulled by one dog alone (Fig. 1c). In this condition, the length of the rope end accessible on each side was 63 cm. The ‘cooperation’ platform (100 × 1 × 20 cm) with two notches and the ‘distant ends’ rope (285 cm long) could only be successfully pulled with a partner (Fig. 1d). In this condition, the length of the rope end accessible on each side was 62 cm.

### Training

**Fig. 2:**
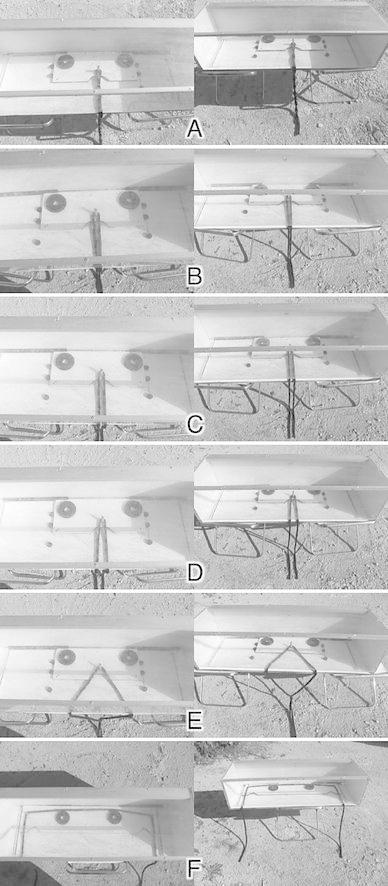
The view of the apparatus used from above (*left column*) and from the front (*right column*) in the different phases of the study: **a** training phase in which the rope ends were entwined for the whole length of the rope, **b** training phase in which the rope ends were positioned close to each other such that they were touching for the whole length of the rope, **c** training phase in which the rope ends were 2 cm apart for the whole length of the rope, **d** training phase in which the rope ends were 5 cm apart for the whole length of the rope, **e** transfer task in which the rope ends were positioned such that close to the box they were far apart (this is where dogs tended to grab the rope) and were close together closer to the ground, **f** testing phase in which the rope ends were so far apart so that one dog alone could not succeed in obtaining the food

Dogs received training trials with the ‘alone’ platform to learn to grab both rope ends simultaneously. Following the training procedure used by Seed et al. (2008), the following training stages were used: rope ends entwined (Fig. 2a), rope ends touching (Fig. 2b), rope ends 2 cm apart (Fig. 2c) and rope ends 5 cm apart (Fig. 2d). Dogs had to successfully pull the platform on three consecutive trials to proceed to the next stage and went back to the previous stage after three consecutive unsuccessful trails. After being successful on the last training phase on three consecutive trials, dogs had to successfully solve this phase again on the next day without going back to the previous phase to ensure that their performance was reliable. Subsequently, dogs were given one transfer task that they had to pass within four trials before proceeding to testing (Table 2). This transfer task presented the rope in a novel way, namely such that in order to successfully obtain the rewards, the dogs had to overcome their usual behaviour, namely grabbing the rope ends close to the base of the box (Fig. 2e). It therefore tested whether or not dogs could generalise from the training trials that both rope ends had to be pulled simultaneously to successfully pull the platform in order to solve the novel transfer test in which the external stimuli differed from the training trials.

**Table 2:**
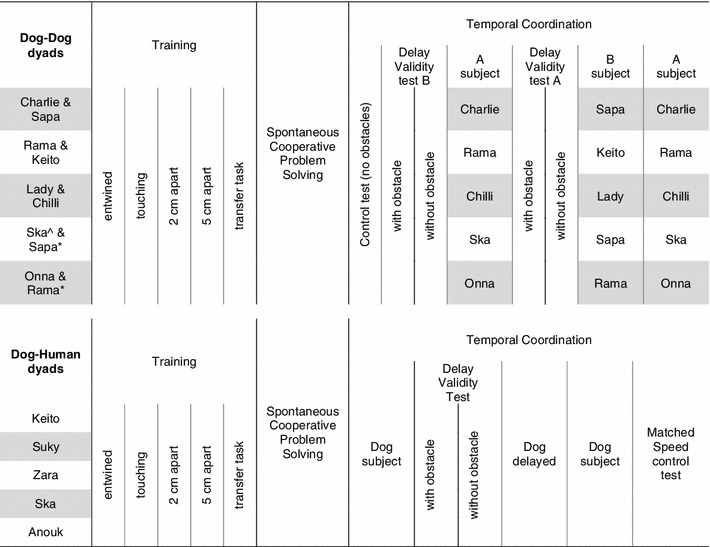
Summary of the different tests that individual dogs participated in training and testing

The different stages of the training are depicted in the order in which they were experienced by the dogs. In the temporal coordination columns for the dog–dog dyads, the delay validity test indicates which of the two dogs in the dyad was given the test at that point (dog A or dog B), and the information which dog was randomly assigned to be in the role of dog A and dog B is given in the columns of ‘A subject’ and ‘B subject’ which indicate which dog was the subject in the respective temporal coordination test. Note that Ska participated in the dog–dog dyad tests after she participated in the dog–human dyad (as denoted by ^^^), and Sapa and Rama participated in the dog–dog dyad tests twice (second time denoted by *). Given that these three dogs already once participated in a delay validity test, they were not tested a second time. The second time Sapa and Rama were tested with a dog (denoted by *) both dogs acted as dog B, because they already had experience of the maze

Dashed lines indicate conditions the order of which was counterbalanced across dogs

### Dog–dog dyads

#### Experiment 1: spontaneous cooperative problem-solving

**Fig. 3:**
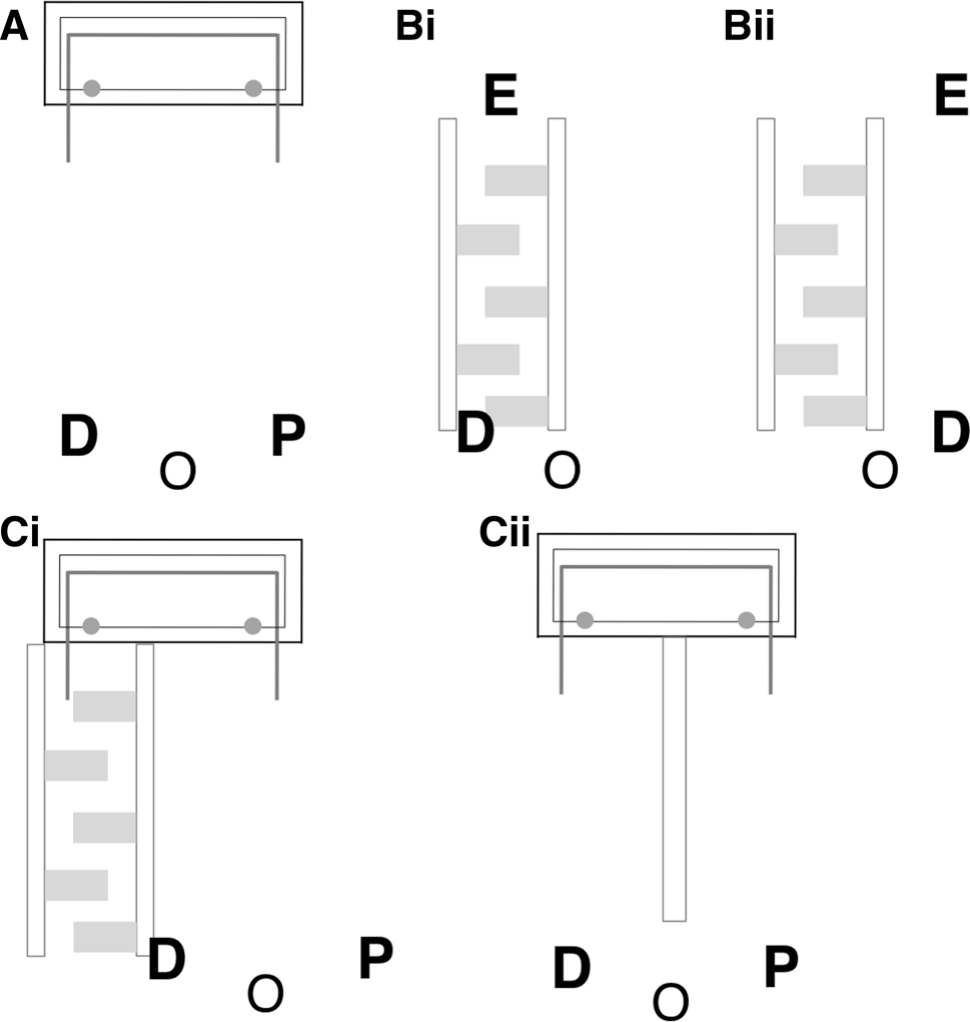
Experimental set-up and the starting positions of the dogs (D) held by their owners (O) and the partner (P) in the **a** *spontaneous cooperative problem*-*solving,* the *maze validity* test with the conditions in which the dog (D) approaches the experimenter (E) **bi** through the obstacles and **bii** directly, as well as the **ci** *delay test* and **cii** *control test*

At the start of each trial, both dogs were either lying down or sitting whilst being held by their owner or an assistant 2.5 m from the apparatus (Fig. 3a). The experimenter positioned the rope ends such that they were 60 cm apart (Fig. 2f), opened the back of the box, baited each notch of the ‘cooperation’ platform with three pieces of ham and closed the back of the box. The position of the dogs was pseudo-randomised such that no dog was on the same side of the apparatus for more than two consecutive trials. Both dogs were released simultaneously. Trials ended either after dogs successfully obtained the rewards, a dog pulled one rope end such that the other end was out of reach for the partner or after 2 min. Dogs were given a maximum of 60 trials or until they successfully solved the task on 20 trials in total. The reasoning behind 20 successful trials in total was to ensure that variation in performance between the different dyads in subsequent tests (especially concerning the dogs’ latencies to approach and pull) could not be explained by differences in reinforcement for having pulled the rope end together with a partner.

#### Experiment 2: temporal coordination

To test whether dogs were capable of temporal coordination, the dogs’ performance when both partners could access the apparatus simultaneously was compared to a delay condition, in which one of the partners was delayed in their ability to approach the rope. The basic set-up and procedure were the same as in Experiment 1, except for the following changes. Two metal fences (360 cm long and 72 cm high) were positioned one in the middle, separating the dogs (in the middle of the apparatus), and the other on the outer left side (Fig. 3ci). Between the two metal fences, barriers made out of bricks (50 × 19 × 19 cm) were positioned to form a ‘maze’ that provided a physical obstacle to slow down one dog's approach to the apparatus. Immediately before a dog was in the role of the delayed partner for the first time, they were given training in overcoming the obstacle and a test investigating how much they were slowed down by it. For this delay validity test, the experimenter stood at the end of the ‘maze’, showed the dog food held in their hand and called the dog’s name. Each dog was given five trials with (Fig. 3bi) and five trials without (Fig. 3bii) the obstacle (order counterbalanced across dogs). Within a dyad, the first dog (A) and the second dog (B) to be the ‘non-delayed’ individual were chosen at random. Dogs were given three tests, with dog A being the non-delayed individual twice in total. Additionally, dogs were tested in a control test in which the two metal fences separated the dogs but in which there were no obstacles such that both dogs could approach the apparatus without any impediment (Fig. 3cii). Here, the position of dogs was pseudo-randomised such that no dog was on the same side on more than two consecutive trials. The order in which dogs experienced these tests is given in Table 2.

### Dog–human dyads

#### Experiment 3: spontaneous cooperative problem-solving

The basic set-up and procedure were the same as in Experiment 1 except for the following changes. The experimenter baited the dog’s side only, and after closing the back of the box, they walked towards the owner (Fig. 3a). From there, the experimenter approached the apparatus in a straight line within 5 s, starting at the same time the dog was released. If the rope end was still available to them, the experimenter held it for a maximum of 10 s. The positions of the dog and human were pseudo-randomised such that the dog was never on one side on more than two consecutive trials. Dogs were given a maximum of 60 trials or until they completed 20 successful trials in total.

#### Experiment 4: temporal coordination

To test whether dogs were capable of temporal coordination with the human partner, dogs’ performance when both dogs and the human partner could access the apparatus simultaneously was compared to when the human was delayed in their approaching (Fig. 3ci). The dog was always in the role of the non-delayed individual first (A). The human partner, who was therefore always delayed first, needed on average 15.6 s (±0.3) to overcome the obstacles. This length of delay was chosen as this was how long it took the experimenter to walk as slowly as possible through the maze, but without stopping her movements, and one of the aims of the experiment with the human partner was to provide a noticeable delay of the partner to the dogs. When the dogs were delayed (B), the human partner approached their rope end directly within 3.4 s (±0.1). As in Experiment 3, immediately before being delayed (B), dogs were given training in overcoming the obstacles and a test investigating how much they were slowed down by it (Fig. 3bi, bii). Additionally, after all delay tests were conducted, dogs were tested in a control when neither dogs nor the human partner was delayed. In this test, the human partner matched the speed of the dogs in approaching the apparatus (matched speed control, Fig. 3cii). The order in which dogs experienced these tests is given in Table 2.

### Analysis

Data were analysed using SPSS 18.0. Where appropriate, data were analysed using repeated-measures ANOVAs, and the graphs accompanying these analyses show means and standard errors of the mean. For within-subject comparisons, the standard errors of the mean were calculated using Cousineau’s (2005) method, which controls for between-subject variation. Data on latencies to approach and pull were composed of mean latencies across all trials within a test for each dog. Twenty per cent of videos were coded by a second rater. Interobserver reliability measures for the coded behaviours were as follows: dog–dog dyads—latency to approach: Pearson’s *r* = 0.83, latency to pull: *r* = 0.80; and dog–human dyads—latency to approach: *r* = 0.92, latency to pull: *r* = 0.91. Where appropriate, exact nonparametric statistics were calculated, and the graphs accompanying these analyses show box plots. Alpha was set at 0.05, and *p* values between 0.05 and 0.1 were interpreted as trends. Unless otherwise specified, all tests were non-directional (two-tailed *p* values).

## Results

### Training

Eleven out of twenty-nine dogs successfully completed the training and solved the transfer task within four trials (for example trials, see Online Resource 1–4). Eight of the eleven dogs solved the transfer task on their first trial (Table 2; for example trial, see Online Resource 5). Two dogs (Charlie and Rama) solved these trials by opening their mouth widely and grabbing both rope ends at the same time. The remaining nine dogs had the same strategy when the rope ends were close together, but when they were 5 cm apart, these dogs tended to pull lightly on each of the rope ends separately or move them with their paw until both rope ends were close together and then pulled stronger and successfully solved the task.

### Dog–dog dyads

#### Experiment 1: spontaneous cooperative problem-solving

All dyads successfully solved the task within 60 trials (Table 2). The following behavioural pattern was observed in four out of five dyads. One of the two dogs in each of these dyads showed a side bias after the first successful trial. The dog kept going to the same rope end which it had pulled on the first successful trial even if that meant that on the current trial it had to cross over to the other side of the apparatus. The other dog (which had approached the apparatus in a straight line) subsequently went across to the free rope end. Eventually, both dogs would approach in a straight line and pull the rope end closest to them. Both dogs in the fifth dyad approached the apparatus in a straight line on all trials (Table 3).

**Table 3:** Individual performance data

Dog	Total number of trials	Trial on which transfer task solved
A: Training		
Charlie	101	1
Sapa	62	4
Keito	24	1
Suky	153	2
Rama	16	3
Ska	41	1
Chilli	26	1
Zara	31	1
Lady	26	1
Anouk	54	1
Onna	128	1

Bi: Dog–dog dyads			Bii: Dog–human dyads		
Dyad	Total number of trials	First successful trial	Dog	Total number of trials	First successful trial
B: Spontaneous cooperative problem-solving					
Charlie and Sapa	22	3	Keito	22	1
Rama and Keito	26	1	Suky	28	2
Lady and Chilli	35	12	Zara	34	1
Ska^^^ and Sapa*	29	1	Ska	27	1
Onna and Rama*	48	15	Anouk	25	1

Ci: Dog–dog dyads					Cii: Dog–human dyads				
Dog	Latency to approach		Latency to pull		Dog	Latency to approach		Latency to pull	
	S	U	S	U		S	U	S	U
C: Temporal coordination									
Charlie	1.2	1.3	0.5	0.6	Keito	5.5	0.9	5.5	1.0
Sapa	1.6	0.4	2.5	1.2	Suky	12.1	1.6	13.5	1.8
Rama	0.1	0.0	0.0	−0.1	Zara	0.9	0.8	1.8	0.8
Lady	−0.5	−1.0	0.2	−0.3	Ska	−0.2	−0.2	0.5	0.2
Chilli	0.4	2.9	0.3	−0.3	Anouk	13.1	2.6	12.9	2.2
Ska	2.2	0.1	2.2	0.1					
Onna	0.1	0.0	0.2	n/a					

(A) performance in the training stage and the transfer task, (B) performance in the cooperative task when paired with (Bi) a conspecific and (Bii) a human partner, (C) latencies to approach and pull the rope end in the delay task relative to the control task in successful and unsuccessful trials when (Ci) in the role of the ‘non-delayed’ dog in dog–dog dyads and when (Cii) paired with a delayed human partner

*S* successful trials, *U* unsuccessful trials

* Dogs that participated in the experiment in a dog–dog dyad for the second time

^^^Dogs that participated in the experiment in a dog–human dyad first

#### Experiment 2: temporal coordination

The delay validity tests assessed whether dogs were slowed down in their approach by the ‘maze’ obstacles. These tests showed that dogs approached the experimenter after 2.0 s (±0.1) without obstacles and after 4.1 s (±0.1) when approaching through the obstacles. Thus, they were successfully delayed by the ‘maze’ (paired *t* test, *n* = 11, *T*(10) = 14.69, *p*_one-tailed_ < 0.001), although the average duration of the delay was short, 2.2 s (±0.1).

**Fig. 4:**
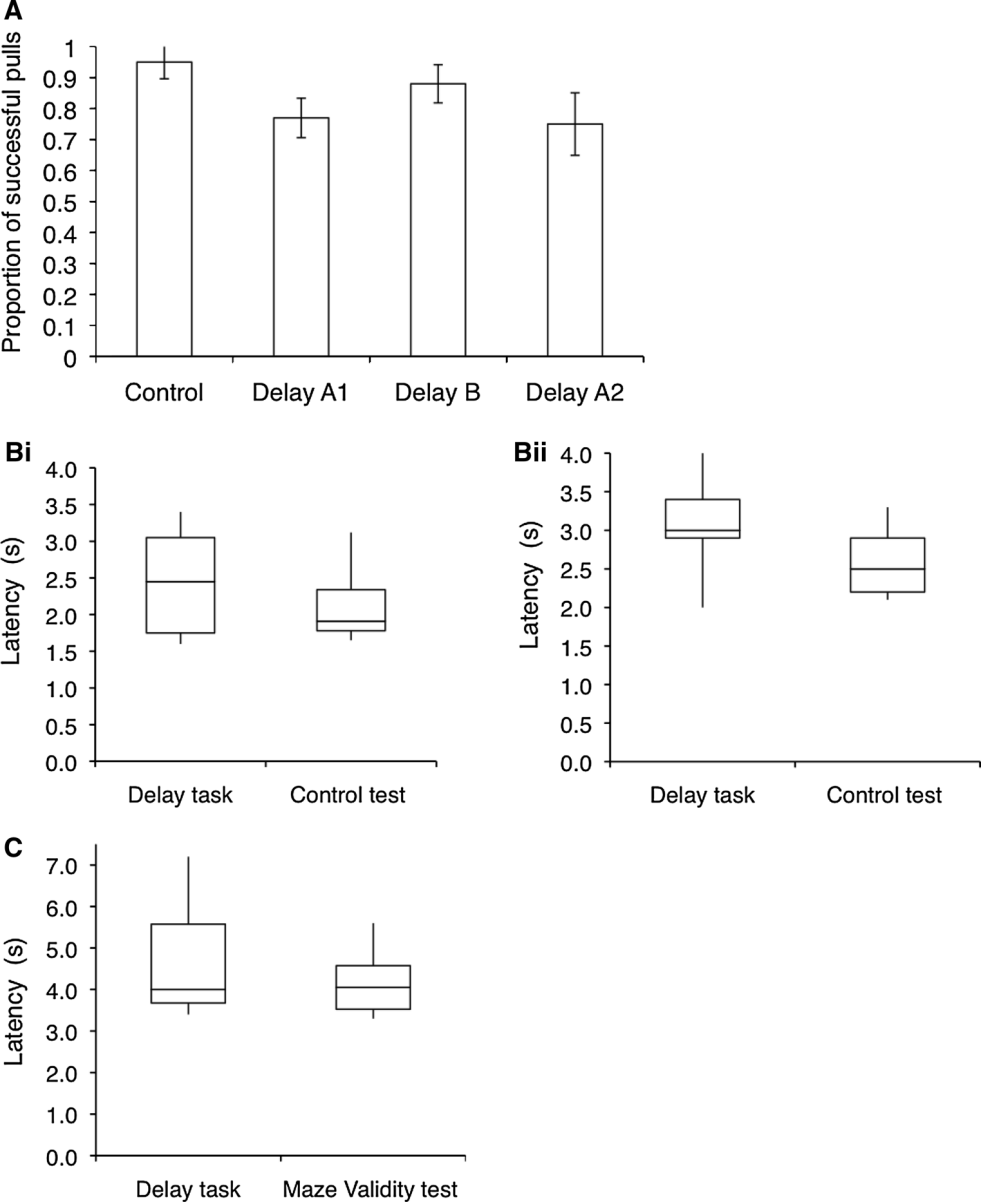
**a** Mean (±SEM) proportion of successful pulls out of total pulls in the control test in which neither dog was delayed and the three delay tests in which one dog was delayed. **b** Average latency of the non-delayed dog to approach the apparatus (**i**) and pull the rope end (**ii**) across all three delay tests and in the control test in which neither dog was delayed. **c** Average latency of the delayed dog to approach the apparatus across the three delay tests and in the maze validity test. *Boxes* show the median and upper and lower quartiles (75 and 25 %) of the data, and the whiskers show the maximum and minimum values

Overall, the success rates in the three delay tests were high (A1: 77 % ± 6; B: 88 % ± 7; A2: 75 % ± 9) and did not differ from each other or the control test (95 % ± 3, repeated-measures ANOVA, *delay test*, *F*_3,12_ = 1.275, *p* = 0.327; Fig. 4a). When in the role of the non-delayed individual, dogs approached the apparatus on average after 2.5 s (±0.3) and pulled the rope after 3.1 s (±0.2) in the delay tests and approached the apparatus after 2.1 s (±0.2) and pulled the rope after 2.6 s (±0.2) in the control test. Thus, across all three delay tests, when in the role of the non-delayed individual, dogs’ latencies to approach were the same as in the control test (exact Wilcoxon signed-rank test, *n* = 8, *T* = 10, *p*_one-tailed_ > 0.05; Fig. 4bi), but the latencies to pull the rope end were longer than in the control test (exact Wilxocon signed-rank test, *n* = 8, *T* = 4.5, *p*_one-tailed_ < 0.05; Fig. 4bii). When successful (for example trial, see Online Resource 6) and unsuccessful trials (for example trial, see Online Resource 7) were analysed separately, longer latencies to pull in the delay tests relative to the control test were only shown for successful trials (exact Wilcoxon signed-rank test, successful trials: *n* = 7, *T* = 1, *p*_one-tailed_ < 0.05, unsuccessful trials: *n* = 7, *T* = 9, *p*_one-tailed_ > 0.05).

The effects of learning in those dogs that were the first non-delayed individual (delay test A1) and were then retested after having been the delayed individual (delay test A2) were investigated using repeated-measures ANOVAs with *delay test* (A1 vs. A2) and *trial order* (1st vs. 2nd half of trials) as within-subject factors. The non-delayed dogs’ latencies to approach relative to the control test showed a decrease from delay test A1 to delay test A2 (repeated-measures ANOVA, *n* = 5, *test*: *F*_1,4_ = 9, *p* = 0.04), but there were no learning effects within a test (*trial order*: *F*_1,4_ = 0.416, *p* = 0.554; *delay test* × *trial order* interaction: *F*_1,4_ = 0.192, *p* = 0.684).

The non-delayed dogs’ latencies to pull relative to the control test did not differ between the A1 and A2 delay tests (repeated-measures ANOVA, *n* = 5, *delay test*: *F*_1,4_ = 1.351, *p* = 0.310), but there was a trend for pulling later in the second half of trials (*trial order*: *F*_1,4_ = 4.610, *p* = 0.098), and this pattern was the same for both tests (*delay test* × *trial order* interaction: *F*_1,4_ = 1.553, *p* = 0.281).

A potential difference between the behaviour of those dogs that were the non-delayed individual for the first time without any experience of being delayed (delay test A1) and those who were the non-delayed individual for the first time but have been the delayed dog before (delay test B) was assessed using repeated-measures ANOVAs with *trial order* (1st vs. 2nd half of trials) as a within-subject factor and *delay test* (A1 vs. B) as a between-subject factor. The dogs’ latencies to approach relative to the control test did not differ between the delay tests A1 and B (repeated-measures ANOVA, *n* = 5, *delay test*: *F*_1,4_ = 1.408, *p* = 0.274), and there were no learning effects within a test (*trial order*: *F*_1,4_ = 0.580, *p* = 0.471; *delay test* × *trial order* interaction: *F*_1,4_ = 0.002, *p* = 0.962). The dogs’ latencies to pull relative to the control test did not differ between the delay tests A1 and B (repeated-measures ANOVA, *n* = 5, *delay* *test*: *F*_1,4_ = 2.635, *p* = 0.149), and there were no learning effects within a test (*trial order*: *F*_1,4_ = 0.040, *p* = 0.848; *delay test* × *trial order* interaction: *F*_1,4_ = 0.134, *p* = 0.726).

The difference between the behaviour of dogs that were the non-delayed individual for the first time (delay test B) and those that were the non-delayed individual for the second time (delay test A2), both after they have had previous experience of being the delayed partner, was investigated using repeated-measures ANOVAs with *trial order* (1st vs. 2nd half of trials) as a within-subject factor and *delay test* (B vs. A2) as a between-subject factor. The dogs’ latencies to approach relative to the control test did not differ between the delay tests B and A2 (repeated-measures ANOVA, *n* = 5, *delay test*: *F*_1,4_ = 1.429, *p* = 0.271), and there were no learning effects within a test (*trial order*: *F*_1,4_ = 0.100, *p* = 0.761; *delay test* × *trial order* interaction: *F*_1,4_ = 0.139, *p* = 0.720).

Finally, when in the role of the delayed partner, dogs’ latencies to approach were the same as in the delay validity tests (Fig. 4c; exact Wilcoxon signed-rank test, *n* = 8, *T* = 14.5, *p* > 0.05), and this pattern was the same for both successful and unsuccessful trials (exact Wilcoxon signed-rank test, successful trials: *n* = 8, *T* = 12.5, *p* > 0.05; unsuccessful trials: *n* = 8, *T* = 8, *p* > 0.05).

### Dog–human dyads

#### Experiment 3: spontaneous cooperative problem-solving

All dogs successfully solved the task with the human partner within 60 trials. Individual performance data are given in Table 2.

#### Experiment 4: temporal coordination

**Fig. 5:**
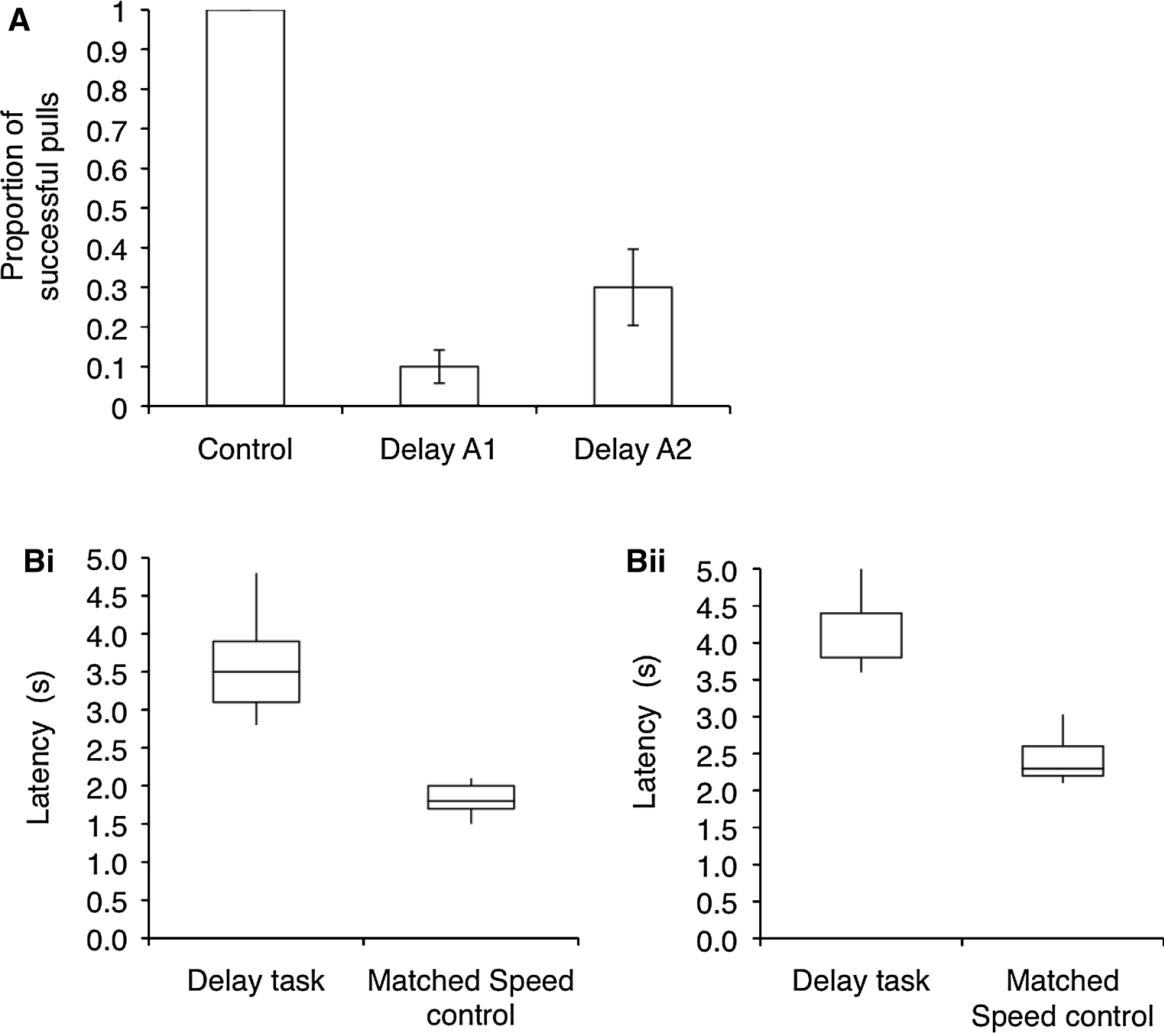
**a** Mean (±SEM) proportion of successful pulls out of total pulls in the control test in which neither partner was delayed and the two delay tests in which the human partner was delayed; **b** average latency of the non-delayed dog to approach the apparatus (**i**) and pull the rope end (**ii**) across all two delay tests and in the matched speed control test in which neither the dog nor the human partner was delayed. *Boxes* show the median and upper and lower quartiles (75 and 25 %) of the data, and the whiskers show the maximum and minimum values

Overall, the dogs were successful on 20 % (±7) of trials when the human partner was delayed 15.6 s (±0.3) and on 100 % of trials in the control test, in which neither the human partner nor the dogs were delayed. Thus, a lower proportion of successful pulls was shown in the delay task than as compared to when no partner was delayed (Wilcoxon signed-rank test, *n* = 5, *T* = 0, *p*_one-tailed_ = 0.05). The proportion of successful pulls increased from the first (A1: 10 % ± 3) to the second (A2: 30 % ± 3) delay test, but there were no learning effects when comparing the first and second half of trials within a test (Fig. 5a; repeated-measures ANOVA, *delay test*: *F* = 10.67, *p* = 0.031; *trial order*: *F* = 3.08, *p* = 0.154; *delay test* × *trial order* interaction: *F* = 0.19, *p* = 0.688).

When the human partner was delayed, dogs approached the apparatus after 3.6 s (±0.1) and pulled the rope after 4.2 s (±0.1). When neither the dog nor the human partner was delayed (control test), dogs approached the apparatus after 1.8 s (±0.1) and pulled the rope after 2.4 s (±0.1). Across both delay tests, dogs approached the apparatus later and pulled the rope end later in the delay tests than in the control test (exact Wilcoxon signed-rank test, approaching: *n* = 5, *T* = 0, *p*_one-tailed_ = 0.05, Fig. 5bi; pulling: *n* = 5, *T* = 0, *p*_one-tailed_ = 0.05, Fig. 5bii).

When successful (for example trial, see Online Resource 8) and unsuccessful (for example trial, see Online Resource 9) trials were analysed separately, there was a trend for latencies to approach and pull in the delay tests to be longer than in the control test for both successful and unsuccessful trials, but this effect was stronger on successful trials (exact Wilcoxon signed-rank test, successful trials: *n* = 5, *T* = 1, *p*_one-tailed_ < 0.10, unsuccessful trials: *n* = 5, *T* = 1, *p*_one-tailed_ < 0.10; successful trials vs. unsuccessful trials: *n* = 5, *T* = 0, *p*_one-tailed_ = 0.05).

Effects of learning on the dogs’ performance between being the non-delayed individual the first time, without having had experience of being delayed themselves (delay test A1) and being the non-delayed individual after having been delayed themselves (delay test A2) were assessed using repeated-measures ANOVAs with *delay test* (A1 vs. A2) and *trial order* (1st vs. 2nd half of trials) as within-subject factors. The dogs’ latencies to approach relative to the control test did not differ between the two delay tests, and there were no learning effects (repeated-measures ANOVA, *n* = 5, *delay test*: *F*_1,4_ = 0.05, *p* = 0.84; *trial order*: *F*_1,4_ = 1.65, *p* = 0.268; *delay test* × *trial order* interaction: *F*_1,4_ = 1.20, *p* = 0.334). Dogs’ latencies to pull the rope end did not differ between the two delay tests (repeated-measures ANOVA, *n* = 5, *delay test*: *F*_1,4_ = 0.58, *p* = 0.491), but latencies to pull were longer in the second half of trials (*trial order*: *F*_1,4_ = 5.01, *p* = 0.089), and this pattern was the same for both delay tests (*delay test* × *trial order* interaction: *F*_1,4_ = 0.03, *p* = 0.87).

## Discussion

All of the dogs that successfully learnt the functionality of the rope-pulling apparatus spontaneously solved the cooperative problem-solving task both when paired with a conspecific and when paired with a human partner. In the delay task, dogs were highly successful when paired with another dog. This success appears to not have been the result of the ‘delayed’ dog trying to approach faster, but the result of the ‘non-delayed’ dog increasing its latency to pull the rope end. When paired with a human partner, the ‘non-delayed’ dogs’ success rates in the delay task were lower, probably due to the human partner being delayed for a longer time. The dogs’ latencies to approach the apparatus and pull the rope end when the human partner was delayed were longer than when neither human nor the dog was delayed. Thus, these findings indicate that dogs were sensitive to the partner’s behaviour and might have inhibited pulling their rope end until their partner was also able to pull the rope.

The delay tasks were conducted to test the necessity for temporal coordination with the partner by introducing a physical obstacle that one of the partners had to overcome, causing them to be delayed in their approach to the apparatus. In the dog–dog task, this delay turned out to be very short (on average 2.16 s) and the dyads showed high success rates that did not differ from a control test in which neither dog was delayed. However, success was dependent on the non-delayed dog inhibiting pulling. In the dog–human delay task, the human partner’s delay was longer (on average 15.6 s) and the success rates were lower than in the control test. Again, success depended on dogs inhibiting pulling the rope end. Although the latencies to pull by the non-delayed dogs overall were relatively short, the average latency to pull on successful trials in the dog–human cooperation for two out of the five dogs exceeded 15 s (Ska and Suky; Table 2). Thus, although inhibiting the necessary action was clearly not easy for dogs, some of them showed a higher inhibitory control than others. Dogs have been found to exhibit problems with inhibitory control in several different tasks (Wobber and Hare 2009; Bray et al. 2013). Interestingly, studies on inhibitory control in dogs have revealed parallels to inhibitory control in humans: difficulties in inhibiting an action were more pronounced in aged individuals (Tapp et al. 2003), and exercising self-control on one task led to decreased motivation to exert inhibitory control on a subsequent task that could be prevented by intake or a taste of glucose (Miller et al. 2010; Molden et al. 2012). These findings suggest that inhibitory control in humans and dogs, and thus likely other non-human animals, might rely on similar mechanisms (Miller et al. 2010). Therefore, effects that have been found to facilitate inhibitory control in humans, such as training of self-control on one task that increases the subsequent performance on a different task (Oaten and Cheng 2006a, b), might present an interesting issue to take into account in future studies on cooperative problem-solving abilities of dogs.

Whilst our results show that dogs used the partner’s behaviour to predict when pulling their string end will be successful, this social cue might not have been the *only cue* used by dogs to solve the task. Theoretically, the cue of seeing the free end of the rope move (when the partner took hold of it) could have been used in the dog–dog tests but not in the dog–human tests, because the human experimenter never pulled the rope but held it, such that no movement occurred on the other end. The length of the rope used in the delay tasks permitted dogs to pull on it a little without bringing the other rope end out of reach for their partner. Thus, dogs could have used the cue of feeling resistance on the rope when pulling to predict when this action would be successful (see also Plotnik et al. 2011). Interestingly, the possibility that individuals might pull the string a little, and then, if this resulted in bringing the reward closer to them, pull more, has not been discussed in the previous studies on cooperative problem-solving. The visual feedback of *seeing rewards incrementally move closer* has previously been shown to act as conditioned reinforcement in two problem-solving tasks in corvids. New Caledonian crows (*Corvus moneduloides*) were found to perform better in a string-pulling task when they had unrestricted visual access to the meat reward attached to the string end and thus could see it moving towards them as the result of their pulling action (Taylor et al. 2010). Similarly, in a ‘water-raising’ task that requires individuals to drop stones into a tube filled with water in order to raise the water level and thus move a floating reward into reach, Eurasian jays (*Garrulus glandarius*) required the feedback of rewards moving closer to themselves to successfully obtain the rewards (Cheke et al. 2011). In the current study, the technique used by all but two dogs (Charlie and Rama) makes it likely that most subjects have partly relied on this sort of feedback to adjust their pulling during training and the transfer task. These dogs pulled lightly until both rope ends were close enough—and possibly, until they saw that pulling resulted in movement of the platform towards themselves—after which they pulled stronger and obtained the rewards. It is likely that the same sort of feedback was used by the dogs in the delay task. Support for this explanation comes from the fact that in the dog–human delay task dogs occasionally (Zara: 2x; Keito: 11x; Anouk: 15x) pulled their string end lightly and then either stood near it or walked away before pulling it again. However, this non-social cue alone cannot explain the dogs’ performance. Critically, dogs’ latencies to pull the string *for the first time* were longer in the delay than in the control tests. This means that dogs must have also anticipated that a delay *before* pulling would yield a successful outcome. Note that these two cues are related, as the partner’s behaviour in the delay task is likely to have been the most salient cue predictive of *when* pulling was likely to result in the rewards moving closer. The assessment whether dogs can learn to coordinate with a partner by only using the partner’s behaviour as a cue will require a test in which the rope is shorter such that already one pull by the subject dog would pull the other end out of reach for their partner.

Both when paired with a conspecific and when paired with a human partner, the dogs were able to spontaneously solve the task within a few trials. In the delay tasks, some effects of learning over the course of all trials were evident within a test, suggesting that dogs’ inhibition of the necessary action was facilitated by learning within a session. However, latencies to approach the apparatus stayed the same throughout testing, suggesting that the dogs’ motivation to participate in the task stayed the same and that increase in successful pulling towards the end of a test might have been due to an increased ability to inhibit pulling the string. This could be the case if it was the dogs’ motivation for the food rather than for participating in the task that decreased throughout a session. The comparisons between the different delay tests suggest that having personally experienced the delay by having to overcome the obstacle was not necessary to show the effect of ‘waiting’ for the delayed partner.

In summary, dogs rapidly generalised the rule learnt during training to perform the necessary action to solve the cooperative task with another individual, even when they were required to adjust their behaviours temporally. Thus, our study extended the previous findings that dogs could solve a task with a conspecific (Bräuer et al. 2013) by showing that they solved a more complicated cooperative task by attending to a social cue, namely their partner, both when this partner was another dog and when it was a human. Although the methodological differences between the dog–dog and dog–human experiments limit a direct comparison, the dogs’ behaviour did not indicate that they perceived the social partner in the cooperative task differently depending on whether it was a conspecific or a human and thus may have been relying on the same representational system in both situations. Similarly, Hare and Tomasello (1999) showed that dogs cannot only utilise human cues to locate hidden food but could also successfully use signals given by conspecific. In the case of cooperative problem-solving, it is yet not clear whether the dogs’ ability to solve such tasks arises from group hunting shown in other social carnivores and, in particular, in wolves, or from abilities evolved during domestication.

Although wolves hunt in groups, there is no consensus as to whether this behaviour is based on coordination, namely whether wolves adjust their actions to one another in relation to the prey. It has been suggested that, aside from the founding pair bond, wolves do not spend enough time alongside other conspecifics to develop such a flexibility of hunting strategies (Mech 1995; Miklósi 2009). Apart from a pilot study, in which two captive wolves successfully solved a cooperative task (Möslinger, unpublished work), experimental studies investigating the cognitive performance of wolves in a cooperative task are still outstanding. The performance of another social carnivore, hyenas, has been claimed to be based on coordinated actions (Drea and Carter 2009), but this interpretation might be hindered by the fact that the delay task used to assess temporal coordination between partners did not require inhibition of the necessary action by the ‘waiting partner’. Thus, further research is needed to establish the extent to which cognitive mechanisms control hyena cooperative problem-solving. Implementing similar tasks in future comparisons between wolves, pet dogs and feral dogs could help determine whether the dogs’ cooperative problem-solving abilities might be derived from group hunting common to all social carnivores, or whether domestication has specifically enabled domestic dogs to coordinate actions with a partner.

## Electronic supplementary material

Below is the link to the electronic supplementary material.

Supplementary material 1 (MPG 2708 kb)

Supplementary material 2 (MPG 2426 kb)

Supplementary material 3 (MPG 2100 kb)

Supplementary material 4 (MPG 9002 kb)

Supplementary material 5 (MPG 4998 kb)

Supplementary material 6 (MPG 4952 kb)

Supplementary material 7 (MPG 7818 kb)

Supplementary material 8 (MPG 9454 kb)

Supplementary material 9 (MPG 10672 kb)

Supplementary material 10 (DOC 25 kb)
