# Supplementary material for: Behavioural coordination of dogs in a cooperative problem-solving task with a conspecific and a human partner
Source: Anim Cogn. 2013 Sep 1;17(2):445–59. doi: 10.1007/s10071-013-0676-1 (PMC3920030; doi:10.1007/s10071-013-0676-1)
Supplement: Supplementary file 10 — Supplementary material 10 (DOC 25 kb) [file 10071_2013_676_MOESM10_ESM.doc]

**ESM_1**

Example of a successful training trial with rope ends entangled (Suky)

**ESM_2**

Example of a successful training trial with rope ends touching (Suky)

**ESM_3**

Example of a successful training trial with rope ends 2cm apart (Suky)

**ESM_4**

Example of a successful training trial with rope ends 5cm apart (Suky)

**ESM_5**

Example of successful solving of the transfer task (Suky)

**ESM_6**

Example of a successful trial in the Dog-Dog Temporal Coordination experiment (Lady & Chilli)

**ESM_7**

Example of an unsuccessful trial in the Dog-Dog Temporal Coordination experiment (Charlie & Sapa)

**ESM_8**

Example of a successful trial in the Dog-Human Temporal Coordination experiment (Anouk)

**ESM_9**

Example of an unsuccessful trial in the Dog-Human Temporal Coordination experiment (Zara)
